# American Public Health Association

**Published:** 1886-01

**Authors:** 


### AMERICAN PUBLIC HEALTH ASSOCIATION.

### THE LOMB PRIZE AWARDS, ETC.

The prizes awarded by Capt. Henry Lomb, of St. Louis, for the best essays on certain topics named, were awarded at the recent meeting of the American Public Health Association, in Washington, D. C., as follows: Best essay on "Healthy Homes and Food for the Working Classes," thirty-six competitors. None of the papers were found to fulfill the terms and conditions on which the prize was to be awarded, and the first prize was not given to any one; but the paper bearing the motto "He who secures a healthy home and healthy food for himself and his family does not live in vain," being considered "one of great merit," the second prize was awarded the author, Prof. V. C. Vaughan, of Ann Arbor, Michigan.

Best essay on "The Sanitary conditions and Necessities of School Houses and School Life"—twenty papers submitted. The committee decided that no one of the papers was entitled to the first prize, but awarded the second prize ($200) to Dr. D. F. Lincoln, of Boston.

Best essay on "Disinfection and Individual Prophylaxis Against Infectious Diseases"—nine essays submitted. The first prize was awarded to the paper bearing the motto "Ad Astra Aspera." On opening the envelope bearing the corresponding motto, it was found that Major Geo. M. Sternberg, U. S. A., was the author. The second prize was not awarded.

Best essay on "The Preventable Causes of Disease, Injury and Death in American Manufactories and Workshops, and the Best Means and Appliances for Preventing and Avoiding them" three essays submitted; neither of which was considered worthy of the first prize. The second prize, however, was given to Dr. Geo. H. Ireland, of Springfield, author of the paper bearing the motto "Preston."

On the strength of the liberality of Capt. Lomb, he was, by rising vote, made a life member, an honor heretofore conferred on no one.

President Cleveland was made an honorary member because of expressions of sympathy and interest in the cause of sanitation.

The prizes not awarded amount to $1,900, which Capt. Lomb authorizes to be used in a similar manner at next meeting.

The next convention of the American Public Health Association will be held at Toronto, October, 1886.

Officers: Dr. H. P. Walcott, of Boston, was elected President; the Vice Presidents are Dr. C. W. Covernton, of Toronto, Dr. G. B. Thornton, of Memphis; Secretary, Dr. Irvin A. Watson, of Concord, N. H.; Treasurer, Dr. J. Berrien Lindsley, Nashville.

